# Radiological Features of T1a Renal Cell Carcinoma on Axial Unenhanced Computed Tomography

**DOI:** 10.7759/cureus.36881

**Published:** 2023-03-29

**Authors:** Aiko Gobara, Takeshi Yoshizako, Rika Yoshida, Takashi Katsube, Yuka Ishikura, Tomomi Kamimura, Yasushi Kaji

**Affiliations:** 1 Radiology, Shimane University Faculty of Medicine, Izumo, JPN

**Keywords:** unenhanced computed tomography, renal mass, tumor detectability, t1a, renal cell carcinoma

## Abstract

CT has become a commonly used diagnostic procedure in clinical practice, particularly in emergency healthcare delivery. Accordingly, the increase in CT usage has increased the likelihood of incidental detections (ID) of renal cell carcinomas (RCCs). This article discusses key points and limitations associated with the diagnosis and characterization of T1a RCC (≤4 cm in diameter) and shows how to improvise on the differentiation of T1a RCC with unenhanced CT (UE-CT). We retrospectively reviewed UE-CT findings of cases associated with the histopathologic diagnosis of T1a RCC and examined the discrimination capacity and radiological characteristics with regard to small RCCs (SRCCs). Detection and characterization of T1a RCC based on UE-CT are not easy in many cases due to limitations in CT findings, but there are notable radiological features to facilitate detection and differentiation. The growth pattern is important for the detection of SRCCs. Internal characteristic features (average attenuation, heterogeneity) are useful for the characterization of the RCC. In addition, CT image visualization techniques may help improve the detectability of RCCs on UE-CT. Radiological features are important in detecting SRCCs and facilitating further examination. In this study, we discuss some cases of T1a RCCs and evaluate the radiological characteristics of the tumors seen on UE-CT.

## Introduction and background

Traditionally, unenhanced CT (UE-CT) is pivotal in a wide range of clinical scenarios, such as the assessment of flank pain [1], monitoring of urolithiasis [2], screening for cancer, and the evaluation of various other conditions [3]. UE-CT has also been used to assess various conditions in patients who cannot undergo CT exams with contrast material. As the incidence of renal cell carcinoma (RCC) proportionately rises with age, the incidence of renal masses is also on the rise, especially in the aging population [4]. Along with the increasingly generalized utilization of imaging studies, incidental detections (ID) of T1a RCC (lesions ≤4 cm in diameter, corresponding to the T1a stage in the latest TNM classification [5]) in asymptomatic patients have also increased [6-8].

The analysis of surveillance and epidemiological data for renal cancer has shown that the prevalence of metastasis at presentation and the five-year disease-specific mortality rates display nonlinear sigmoidal relations with the tumor size [9]. Therefore, the detection of RCC at an early stage by axial UE-CT is of significance in various scenarios, such as abdominal screening. Small RCCs (SRCCs) (≤4 cm) may not be identified easily by screening UE-CT since limited information is provided. Accordingly, it is important to be well-versed in the characteristic radiological features and detection limits of SRCCs on UE-CT scans to avoid missing these tumors.

## Review

Key points regarding the detection and differentiation of SRCCs on axial UE-CT

In this review, we focus on the detection of SRCCs by axial UE-CT and the differentiation of RCCs from benign lesions because unintentional diagnosis or findings in the kidney is very usual and many are found to be renal lesions (RL) or masses. A practical approach used to detect and differentiate SRCCs by axial UE-CT involves the following series of key points: tumor contour, tumor attenuation, tumor heterogeneity (Hty) or homogeneity (Hgy), tumor margin, tumor location, tumor size, and the complexity of cystic masses.

Tumor Contour

It is important to check the renal margin for the detection of SRCCs. The renal mass may be identified as a nodule protruding from the outer segment of the kidney. On pathological examination, renal tumor contour can be classified into the following three categories: if more than 50% of the tumor protrudes out of the renal cortex, they are classified as “exophytic (EXT)”; if <50% of the tumor protrudes out of the renal cortex, they are called ‘‘mesophytic (MST)’’; the ones that are completely surrounded by unaffected renal parenchyma (RP) are termed “endophytic (ENT)” [10,11]. 

**Figure 1:**
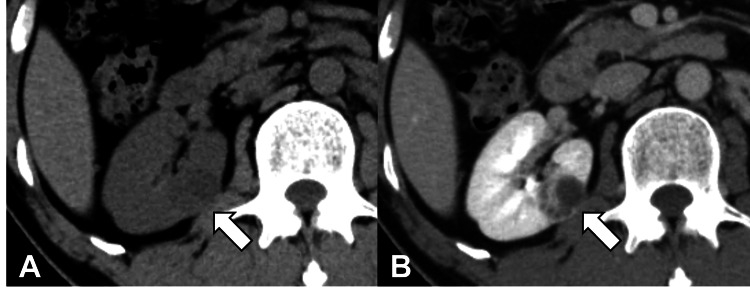
CT images of a 57-year-old woman with endophytic clear cell RCC A: unenhanced image; B: enhanced image in nephrographic phase A heterogeneous renal mass without deformation of the cortical outline is seen in the inner segment of the right kidney on UE-CT (arrows). Pathologic examination confirmed that the mass was a 2.0-cm clear cell RCC of the ENT type ENT: endophytic; RCC: renal cell carcinoma; UE-CT: unenhanced computed tomography

Previous studies have suggested that the detection of SRCCs on UE-CT scans would be strongly influenced by the dimensions of tumor growth [12], with ENT (entire tumors surrounded by uninvolved RP) being tougher to determine compared to MST or EXT (Figure 1).

**Figure 2:**
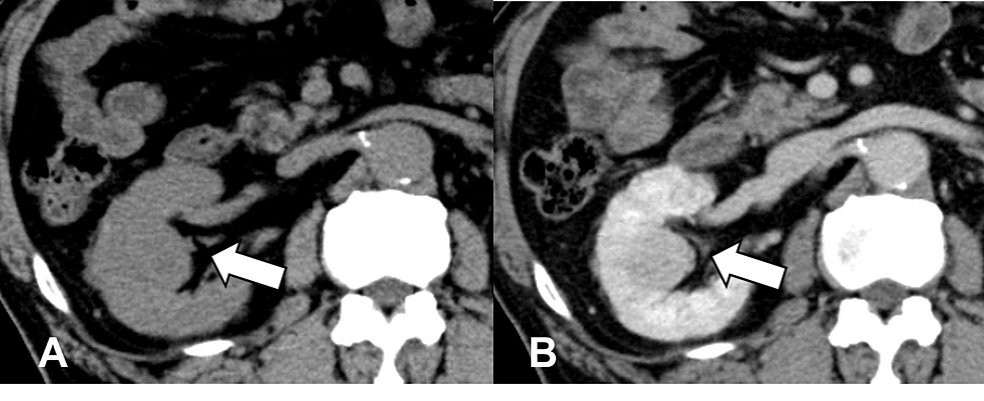
CT images of a 71-year-old man with clear cell RCC A: unenhanced image; B: enhanced image in nephrographic phase On axial UE-CT, a 2.8-cm isodense mass with unclear margins is seen in the right kidney (arrow). The mass protrudes inside the renal cortex like a column of Bertin. Pathologic examination confirmed that the mass was a 2.8 cm clear cell RCC protruding inside the renal cortex RCC: renal cell carcinoma; UE-CT: unenhanced computed tomography

In rare cases, a mass project may reside inside the renal cortex, and it will be difficult to differentiate it from a hypertrophic column of Bertin (Figure 2).

An enlarged column of Bertin is isodense with the normal RP on unenhanced and UE-CT images [13,14]. It is difficult to distinguish RCC that shows homogeneous density from prominent columns of Bertin on UE-CT and it usually requires following up with contrast-enhanced CT or MRI. In such cases, UE-CT has limitations in differentiating RCC. MRI examinations are more sensitive than CT and the hypertrophic column of Bertin displays an enhancement pattern similar to that of normal RP on dynamic MRI [15,16].

Tumor Attenuation

It is important to check tumor attenuation for the identification and differentiation of SRCCs.

A renal mass can be recognized in the presence of different attenuation responses from the RP. Moreover, the specific attenuation associated with renal masses suggests the presence of fat masses and hemorrhagic cysts. It was reported that all proven RCCs in a series contained noncalcified regions with intensities in the range of 20-70 HU on UE-CT [17]. On UE-CT, the presence of regions of interest with attenuations less than −10 HU allows for the confident identification of fat. Detection of intralesional macroscopic fat is essentially diagnostic of angiomyolipoma [18-20]. It was also reported that attenuations >70 HU on UE-CT is a mandatory diagnostic procedure for hemorrhagic or proteinaceous cysts [21].

**Figure 3:**
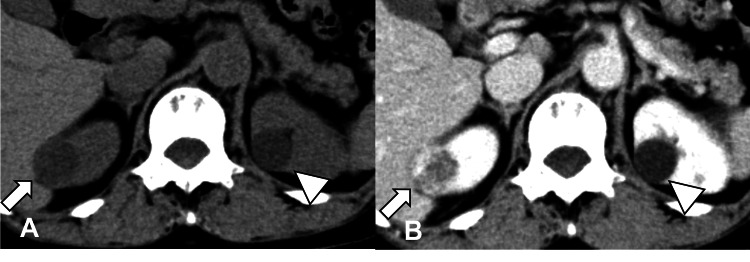
CT images of a 39-year-old man with clear cell RCC A: unenhanced image; B: enhanced image in nephrographic phase On axial UE-CT, there are two hypodense masses with a diameter of 2.1 cm, one in each kidney. Both masses are homogeneous and low-density lesions. The mean attenuation of the right mass is 26 HU (arrow), which is slightly higher than that of the left mass (arrowhead, 18 HU). The right renal mass shows enhancement, which proved to be a clear cell type RCC pathologically RCC: renal cell carcinoma; UE-CT: unenhanced computed tomography

In most cases, renal masses completely outside the 20-70 HU range on UE-CT were found to be benign in previous investigations. Thus, it has been suggested that indeterminate RLs with areas in the 20-70 HU danger zone generally warrant further workups [6] (Figure 3).

Therefore, renal regions with these attenuations should be considered for further imaging studies such as ultrasonography or MRI. If an RL exhibits attenuations similar to those for water (−10 to 20 HU), it is considered to be diagnostic for benign renal cysts. However, a few solid RCCs have been reported that demonstrated attenuations similar to water on UE-CT. However, all of these tumors were heterogeneous, which suggested that their distinction from simple cysts was feasible [17].

Tumor Hty or Hgy

**Figure 4:**
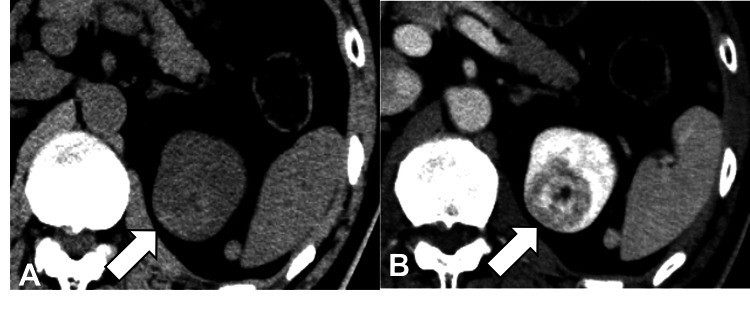
CT images of a 59-year-old man with clear cell RCC A: unenhanced image; B: enhanced image in nephrographic phase On axial UE-CT, a 2.7-cm heterogeneous isodense mass with unclear margins is seen in the left kidney (arrows). The mass protrudes slightly outside the renal cortex and its mean attenuation is 31 HU. It shows heterogeneous enhancement and proved to be a clear cell type RCC pathologically RCC: renal cell carcinoma; UE-CT: unenhanced computed tomography

Assessment of Hty is important for the differentiation of RCC from a benign lesion. Malignancy is undoubtedly the problem with many IDs of renal masses that are heterogeneous on UE-CT scans (Figure 4) [22].

Characteristics that highlight Hty in a renal mass comprise wall hypertrophy, one or more septa, mural nodule(s), measurable or visible attenuation differences, and calcification [23]. Pooler et al. have reported that on UE-CT, out of the 193 pathologically diagnosed RCCs that measured from 1.1 to 20.1 cm in the maximum transverse cross-sectional diameter (MTCD) with a mean value of 5.1 ± 3.4 cm, 90.7% (n=175) were categorized as Hty and 9.4% (n=18) were categorized as Hgy. Furthermore, Hgy lesions showed a remarkably lower diameter compared to the heterogeneous lesions, as complex internal contents may be tougher to discern in smaller lesions [22]. O'Connor et al. reported that on UE-CT, out of the 43 pathologically proven RCCs that ranged in MTCD from 1.0 to 9.7 cm (mean: 3.9 cm), 21 RCCs were categorized as Hty and 22 RCCs as Hgy. Furthermore, no difference was observed in the detection of renal carcinoma on the basis of Hty [24]. Therefore, even in the case of Hgy, there is a possibility of RCC occurrence, and the presence or absence of Hty may not be helpful in the detection of RCCs. However, Hty sometimes provides an important clue for distinguishing an RCC from a benign lesion. Therefore, it is important to identify the Hty.

**Figure 5:**
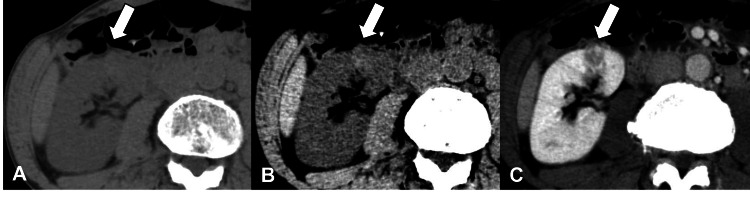
CT images of a 70-year-old man with clear cell RCC A; UE-CT, width: 400 HU, level: 40 HU; B: UE-CT, width: 185 HU, level: 45 HU; C: enhanced image in nephrographic phase On axial UE-CT, a 1.2-cm Hgy low-density mass with unclear margins is detected in the right kidney (arrows). The mass was undetectable on unenhanced scans, but it could be detected by using the ‘‘liver window’’. Pathologic examination confirmed that the mass was a 1.2-cm clear cell RCC RCC: renal cell carcinoma; UE-CT: unenhanced computed tomography

It is considerably tougher to detect and isolate SRCCs with apparent Hgy low-density or isodense tissues in comparison to RP. If an SRCC is Hgy and exhibits an isointense intensity compared to the RP, special image display methods are required to distinguish it on UE-CT. The level of a “liver window” (width: 150 HU, level: 88 HU) setting for isolating SRCCs on UE-CT scans has also been documented [25]. Image display techniques can help overcome the limitations of UE-CT for the isolation and identification of SRCCs (Figure 5).

Tumor Margin

Although there are no published reports showing that RCCs have unclear margins compared to benign lesions, Schieda et al. have reported that while a disrupted pseudo-capsule with an invasive margin was often seen in the sarcomatoid RCC compared to the clear cell RCC group, there was no remarkable difference between them [26]. On UE-CT, the margin of SRCC may sometimes show irregular margins owing to the tumor’s Hty (Figure 4).

Tumor Location

**Figure 6:**
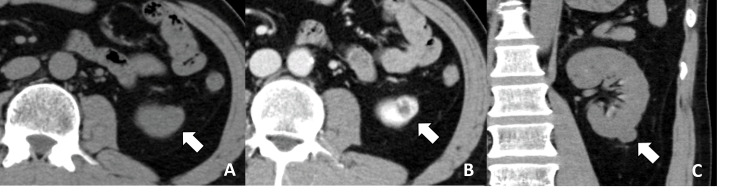
CT images of a 50-year-old man with clear cell RCC A: unenhanced image; B: enhanced image; C: unenhanced coronal image On axial UE-CT, a 1.8-cm heterogeneous isodense mass with unclear margins is seen in the left kidney (arrows). The mass protrudes slightly outside the renal cortex. When multiplanar reconstruction is used, the mass can be detected in the lower pole of the kidney by visualizing the protrusion of the mass on the coronal image RCC: renal cell carcinoma; UE-CT: unenhanced computed tomography

Localization can affect the detectability of SRCCs on UE-CT scans. In particular, masses at the superior or inferior pole of the kidney are sometimes overlooked when only axial UE-CT images are evaluated. It has been reported that it is tough to detect T1a RCCs in the superior or inferior poles of the kidney and/or tumors with isodense intensity compared to the RP based solely on the interpretation of unenhanced axial CT images [27]. If an RCC is located in the superior pole, it must be distinguished from an adrenal mass. Multiplanar reconstruction can help overcome problems related to the tumor location and growth pattern (Figure 6).

Tumor Size

**Figure 7:**
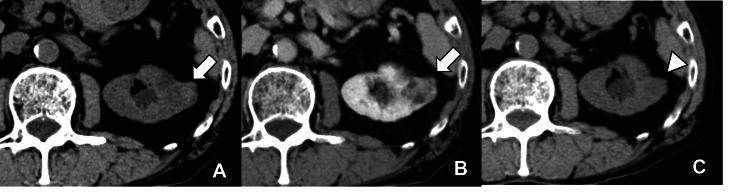
CT images of an 83-year-old man with clear cell RCC A: unenhanced image; B: enhanced image; C: unenhanced image 4 years before A, B: On axial UE-CT, a 2.2-cm heterogeneous hypodense mass is detected in the left kidney and enhanced heterogeneously (arrows). The mass protrudes from the outer segment of the left kidney. Pathologic examination confirmed that the mass was a 2.2-cm clear cell RCC. C: The renal mass was smaller and not more protruded from the outer segment of the left kidney (arrowhead). The mass was homogeneous and of low density on UE-CT, and therefore it was very difficult to be detected as malignancy 4 years before RCC: renal cell carcinoma; UE-CT: unenhanced computed tomography

On UE-CT, the tumor size has a profound effect in terms of locating the RCC. In the analysis of tumor sizes, the area spanned by the receiver operating characteristic (ROC) curve in detecting SRCC on UE-CT was significantly lower than that for large RCC [12,25]. This may be because of the CT attenuation of SRCCs that may reveal Hgy CT attenuation much like the background RP [22]. It was also found that complex internal structures will be highly tough to locate in a small lesion, and greater tumor sizes likely facilitate Hty. Larger tumors show Hty owing to necrosis, hemorrhage, and calciﬁcation [28]. The detection of SRCCs is not straightforward because imaging characteristics are often nondiagnostic owing to the partial volume effect (Figure 7).

The presence of small renal masses should be considered with suspicion for the possibility of RCC.

The Complexity of Cystic Masses

**Figure 8:**
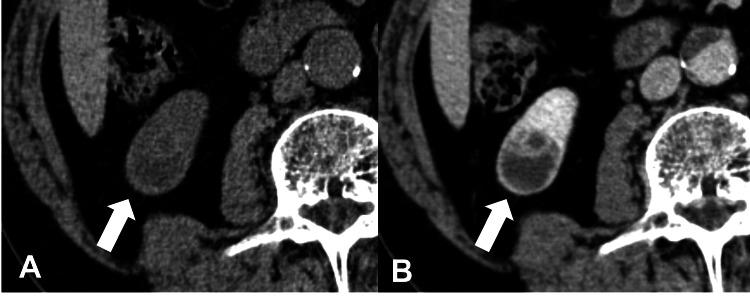
CT images of a 68-year-old man with clear cell RCC A: unenhanced image; B: enhanced image in nephrographic phase On axial UE-CT, a 2.4-cm heterogeneous hypodense mass with a thick wall and a mural nodule is seen in the lower pole of the right kidney (arrow). The enhanced image demonstrates a well-enhancing mural nodule within the mass, suggestive of Bosniak category IV. Pathologic examination confirmed that the mass was a clear cell RCC RCC: renal cell carcinoma; UE-CT: unenhanced computed tomography

The use of the Bosniak classification is recommended for the evaluation of any cystic renal masses [29,30]. Any Hgy mass ranging between −10 and 20 HU on CT on UE-CT has also been considered a simple cyst (Bosniak I) and does not necessitate subsequent investigation. The likelihood of malignancy of cystic renal masses is based on the degree of cyst complexity. On UE-CT scans, cystic RCCs typically show thickened walls and/or mural nodules, whereas simple cysts have paper-thin smooth walls [3]. It was reported that Bosniak category 2F lesions with minimally irregular septae or walls, as well as lesions with an indistinct parenchymal interface, were more likely to exhibit progression to cancer [31]. Cystic RCCs are more likely to have thick and indistinct margins and/or irregular septae compared with benign masses. This finding may provide a clue to the distinction of these lesions from benign lesions on UE-CT (Figure 8).

## Conclusions

As the use of UE-CT has become more common, reports of its usefulness in detecting RCCs and their radiological characteristics have proliferated. However, few studies have evaluated the detection rate of SRCCs on UE-CT. It should be noted that while most adventitious renal masses are benign, most RCCs are identified incidentally. Diagnosing small renal tumors is often difficult, but early diagnosis of RCC at its curable stage is important for a better prognosis. Caution should be exercised in determining whether a renal mass is a benign cyst based only on UE-CT. Accordingly, it is important to have a good knowledge of the properties of SRCCs on UE-CT scans and appropriate CT imaging techniques. However, it is also true that many clues about SRCCs cannot be detected on UE-CT images. Given the current limitations of UE-CT images for detecting and differentiating SRCCs, additional efforts are necessary to improve the utility of UE-CT with regard to the stated purpose.
